# Characterization of two newly isolated *Staphylococcus aureus* bacteriophages from Japan belonging to the genus *Silviavirus*

**DOI:** 10.1007/s00705-020-04749-6

**Published:** 2020-08-03

**Authors:** Naoya Kitamura, Eri Sasabe, Shigenobu Matsuzaki, Masanori Daibata, Tetsuya Yamamoto

**Affiliations:** 1 Department of Oral and Maxillofacial Surgery, Kochi Medical School, Kochi University, Nankoku, Kochi 783-8505 Japan; 2 Department of Microbiology and Infection, Kochi Medical School, Kochi University, Nankoku, Kochi 783-8505, Japan; 3 Present Address: Department of Medical Laboratory Science, Faculty of Health Sciences, Kochi Gakuen University, Kochi, Kochi 780-0955, Japan

## Abstract

Two *Staphylococcus aureus* bacteriophages, KSAP7 and KSAP11, were isolated from sewage and characterized. Based on morphology and DNA sequences, they were assigned to the genus *Silviavirus*, subfamily *Twortvirinae*, family *Herelleviridae*, whose members are hypothesized to be suitable for bacteriophage therapy. The KSAP7 and KSAP11 genomes were 137,950 and 138,307 bp in size, respectively. Although their DNA sequences were almost identical, evidence of site-specific DNA rearrangements was found in two regions. Changes in the number of PIEPEK amino acid sequence repeats encoded by *orf10* and the insertion/deletion of a 541-bp sequence that includes a possible tail-related gene were identified.

**Electronic supplementary material:**

The online version of this article (10.1007/s00705-020-04749-6) contains supplementary material, which is available to authorized users.

Increased resistance of pathogenic bacteria to antibacterial agents has prompted the development of alternatives and/or supplements to current antibacterial therapies. One of the most potent antibiotic-independent alternatives is bacteriophage (phage) therapy [1]. In phage therapy, phages (viruses specific for bacteria) or phage products such as endolysin and depolymerase [2–4] function as antibacterial agents. A large number of phages have been isolated and characterized for the purpose of developing phage therapies. Virulent phages, which do not exhibit a lysogenic cycle, are generally considered more suitable than lysogenic phages due to the low likelihood of superinfection exclusion, in which lysogens act against the same type of phage, and of the transfer of pathogenic genes [5].

Most of the therapeutic candidate phages reported to date have been assigned to the order *Caudovirales* (namely tailed phages) [6, 7]. Phages with a tail and contractile sheath, which have been traditionally assigned to the family *Myoviridae*, have now been reorganized into three families based on DNA sequence: *Myoviridae*, *Herelleviridae*, and *Ackermannviridae* [8].

In this study, we isolated and characterized two previously undescribed *Staphylococcus aureus* phages, KSAP7 and KSAP11, belonging to the genus *Silviavirus*, subfamily *Twortvirinae*, family *Herelleviridae*, whose members are reportedly suitable for phage therapy [9]. Comparison of the DNA sequences of the phages KSAP7 and KSAP11 suggested that there are at least two regions in the phage genome where site-specific DNA rearrangement occurs.

The bacterial strains used in this study are shown in Table S1. Reagents and media were purchased from Nacalia Tesque (Kyoto, Japan), FUJIFILM Wako Pure Chemical Corporation (Osaka, Japan), Sigma-Aldrich Japan (Tokyo, Japan), and BD Japan (Tokyo, Japan).

Phages were isolated from a sewage plant in the city of Kochi, Japan, essentially according to a previously reported method [10, 11]. Briefly, after removing debris from sample water using a loose filter (no. 514A, Advantec, Tokyo, Japan), phages were concentrated by addition of 10% polyethylene glycol (PEG) 6000 and 0.5 M NaCl (approximate final concentration) with successive centrifugation at 10,000 × *g* for 5-10 min at 4 °C. The pellet was resuspended in tryptic soy broth supplemented with 20 mM MgCl_2_ (TSBM) and then filtered using a 0.45-μm-pore-size filter. The filtrate was inoculated with *S. aureus* strain IID975 and incubated overnight at 37 °C to enrich for phages. The culture was centrifuged at 10,000 × *g* for 5-10 min at 4 °C, and the supernatant was filtered. Phages were isolated by three successive single-plaque isolations on *S. aureus* IID975, using the double-layer method with TSBM-agar plates (upper layer, 0.5% agarose; lower layer, 1.5% agarose).

The host range was examined by streak testing on bacterial lawns of each strain to assess the plaque-forming ability of the phages. Briefly, 10 μl of phage suspension was streaked using a disposable plastic loop onto the solidified upper layer with each host, and plaque formation was assessed after incubation for approximately 15 h at 37 °C.

Phages were amplified and purified as described previously for electron microscopic observation and DNA extraction [10, 11]. Briefly, phages were amplified using *S. aureus* strain IID975 as the host in 200-250 ml of TSBM liquid medium at 37 °C. After centrifugation of the culture at 10,000 × *g* for 5-10 min at 4 °C, 10% PEG 6000 and 0.5 M NaCl (approximate final concentration) were added to the supernatant, which was centrifuged at 10,000 × *g* for 20 min at 4 °C. The pellet was resuspended in TM (10 mM Tris-HCl, 5 mM MgCl_2_ [pH 7.2]) containing 100 μg of DNase I and 100 μg of RNase A per ml and incubated for 30 min at 37 °C. The phages were overlaid on a discontinuous CsCl density gradient (ρ [specific weight] =1.3, 1.5, and 1.7) and then ultracentrifuged at 100,000 × *g* for 60 min at 4 °C using S80AT3 and S100AT4 rotors and a GX series Himac CS 100GX micro-ultracentrifuge (Hitachi Ltd., Tokyo, Japan). The phage band was recovered and dialyzed against 1000 ml of AAS (100 mM ammonium acetate, 10 mM NaCl, 1 mM MgCl_2_, and 1 mM CaCl_2_, [pH 7.2]) for 60 min at 4 °C (molecular weight cutoff, 10 kDa). The sample of phages was then placed on a mesh grid (Excel support film, Nisshin EM, Tokyo, Japan), negative-stained with 2% uranyl acetate, and examined by electron microscopy (JEM-1400 Plus, JEOL, Tokyo, Japan). The remaining dialyzed suspension was ultracentrifuged again at 100,000 × *g* for 60 min at 4 °C. Phage DNA was prepared from the pellet by phenol extraction after solubilization in TE containing 1% SDS, followed by ethanol precipitation. The DNA samples were subjected to Illumina sequencing by Eurofins Genomics (Tokyo, Japan). Gapped regions were filled using the primer-walking method with a Model 3130 Genetic Analyzer (Applied Biosystems, Foster City, CA, USA) after sequencing reactions using an ABI Big Dye Terminator 1.1 Cycle Sequencing Kit (Applied Biosystems). The sequence around the *orf10* gene in the KSAP11 genome was determined after cloning of the PCR product encoding this region. The complete DNA sequences of KSAP7 and KSAP11 were submitted to the DDBJ/EMBL/GenBank database under the accession numbers LC492751 and LC492752, respectively.

Host range of the phages was examined using 30 strains, including both MSSA and MRSA strains (Table S1). Although KSAP11 formed plaques on all of the strains examined (30/30), KSAP7 did not form plaques on two strains, MR7 and MR12 (28/30), suggesting that some aspect of the infection process, such as adsorption, differs between KSAP7 and KSAP11.

KSAP7 and KSAP11 were morphologically similar (Fig. 1). KSAP7 had an icosahedral head (108.3 ± 4.3 nm [mean and standard deviation]) and tail with a contractile sheath (length 234.8 ± 1.4 nm; width, 19.2 ± 1.7 nm). Similarly, KSAP11 also had an icosahedral head (103.0 ± 1.5 nm) and tail with a contractile sheath (length 234.6 ± 5.4 nm; width, 20.3 ± 0.7 nm). Means and standard deviations were determined from five and three samples of KSAP7 and KSAP11, respectively, using images at ×80,000 magnification.

**Fig. 1:**
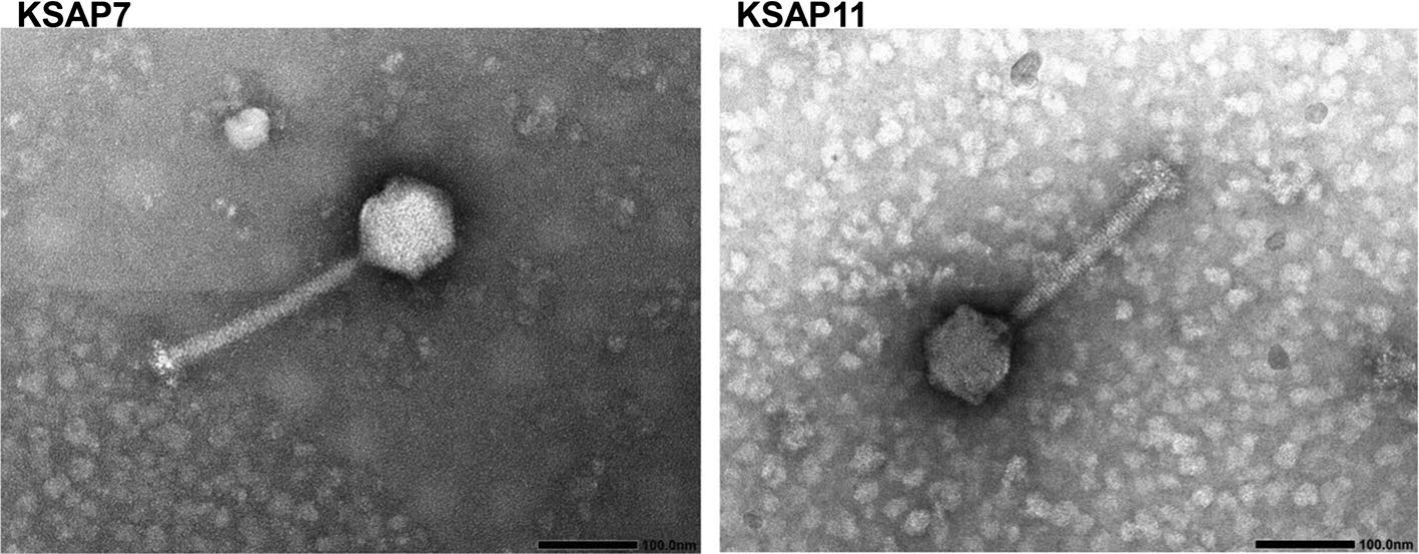
Electron micrographs of phages KSAP7 and KSAP11. Bar, 100 nm. Photographs were taken at ×80,000 magnification

The KSAP7 and KSAP11 genomes were 137,950 bp and 138,307 bp in size, respectively. A BLASTn search (https://blast.ncbi.nlm.nih.gov/Blast.cgi) indicated that these phages were closely related to members of the genus *Silviavirus*, namely SA11 [12], Remus and Romulus [9], Stau2 [13], StAP1 [14], and ϕMR003 [15]. Phylogenetic analysis using MEGA7 software [16] also indicated that KSAP7 and KSAP11 were closely related to these phages, particularly Remus and Romulus (Fig. S1). Based on these morphologic and genetic data, phages KSAP7 and KSAP11 were tentatively assigned to genus *Silviavirus*, subfamily *Twortvirinae*, family *Herelleviridae*.

The KSAP7 and KSAP11 genomes appeared to contain 187 and 188 predicted genes, respectively. In this paper, genes and gene products are designated as *orf* and ORF, respectively. In the genomes of both phages, the regions spanning *orf1* to *orf142* (ca. 113,000 bp) and *orf185* to *orf187* (ca. 1,000 bp) were oriented in the same direction. In contrast, the region spanning *orf143* to *orf184* (ca. 23,000 bp) was oriented in the reverse direction relative to the remaining *orfs* (Fig. 1a). A BLASTp search of the ORFs did not reveal any genes related to lysogeny, suggesting that these are virulent phages. In addition, no genes related to toxins such as hemolysin, enterotoxin, toxic shock syndrome toxin 1, Panton-Valentine leucocidin, and exfoliative toxin, which are known to be present in the *S. aureus* genome and/or in those of its prophages, or drug resistance were found.

In Remus and Romulus phages, genes encoding the large terminase subunit, portal protein, helicase, ribonucleoside reductase subunit, DNA-repair protein, and DNA-polymerase I reportedly split into several parts and/or include a group I intron encoding a homing nuclease gene [9, 17]. The corresponding genes in the KSAP7 and KSAP11 genomes for the large terminase subunit (*orf1* to *orf6*), portal protein (*orf12* to *orf15*), helicase (*orf47* to *orf48*), ribonucleoside reductase subunit (*orf68* to *orf72*), and DNA-repair protein (*orf83* to *orf85*) appeared to be arranged in a manner similar to that reported for the Remus and Romulus genomes. It was predicted that mRNA splicing and/or fusion of the synthesized peptides would occur in the synthesis of each protein.

Although a putative group I intron including the I-RoReV endonuclease gene was identified between the two segmented DNA polymerase I *orfs* in the genome of Remus and Romulus phages, no endonuclease gene sequence was found in either the KSAP7 or KSAP11 genome (*orf77* and *orf78*). However, the intein amino acid sequence region, which is removed after translation, in the DNA polymerase I (ORF75) of Remus and Romulus was also predicted to be present in the DNA polymerase I (ORF78) of KSAP7 and KSAP11 [9]. The presence and type of homing-endonuclease gene and presence of an intein-encoding sequence in this genome region of silviaviruses varied considerably from strain to strain (data not shown).

The DNA sequences of phages KSAP7 and KSAP11 were identical except in two regions (*orf10* and *orf88.1*), which was confirmed by comparison of the two complete nucleotide sequences using BLASTn. Since these phages were isolated from the same sewage sample, they are predicted to have diverged from a common ancestor very recently.

*Orf10* encodes a protein of 286 and 226 amino acid residues in KSAP7 and KSAP11, respectively. The amino acid sequence of the N-terminal region (amino acid residues 1-199) encoded by *orf10* was identical in KSAP7 and KSAP11. Furthermore, the C-terminal region (amino acid residues 260-286) encoded by KSAP7 *orf10* was identical to that (amino acid residues 200-226) encoded by KSAP11 *orf10*. However, the number of repeats of a specific amino acid sequence, PIEPEK, encoded by *orf10* was 12 in KSAP7 and 2 in KSAP11 (Fig. 2b). A search for *orf10*-like genes in other *Silviavirus* phages indicated that the number of PIEPEK repeats varies from 2 to 12 (Table 1). However, *orf10*-like genes of phage K and Twort belonging to the same subfamily (*Twortvirinae*) as KSAP7 and KSAP11 but different genera (*Kayvirus* and *Twortvirus,* respectively) did not have this repeat sequence (Table 1) [8].

**Fig. 2:**
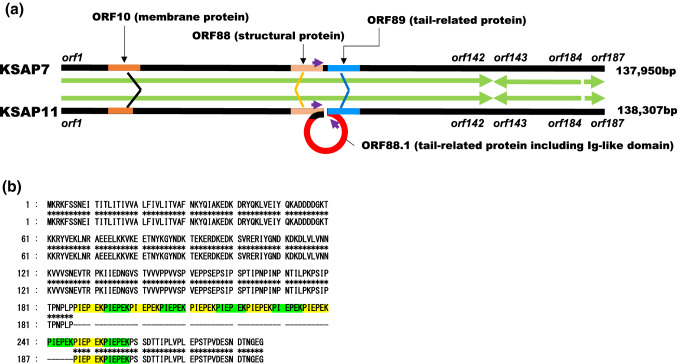
Complete genome analysis of KSAP7 and KSAP11. **a** Two variable regions identified in the phage KSAP7 and KSAP11 genomes. Large green arrows indicate the direction of *orfs,* and small purple arrows indicate a 50-bp direct nucleotide sequence repeat. Insertion/deletion of a 541-bp DNA sequence may occur between *orf88* and *orf89* by recombination involving 50-bp direct repeats according to the Campbell model. **b** Variation in the number of PIEPEK repeats in ORF10. Upper row, KSAP7 ORF10; lower row, KSAP11 ORF10

**Table 1:** Features of two variable regions in the silviavirus genomes

Phage	ORF10-like protein				ORF88.1-like protein		
	Related gene product	Number of amino acid residues	Number of PIEPEK repeats	Identity to KSAP7 ORF10 (%)*	Related gene product	Number of amino acid residues	Identity to KSAP11 ORF88.1 (%)*
KSAP7	ORF10	286	12	100	Absent		
KSAP11	ORF10	226	2	79.02	Present (ORF88.1)	170	100
Remus	Remus_009	277	8	90.2	Present (Remus_086)	170	97.64
Romulus	Romulus_009	277	8	90.2	Present (Romulus_086)	170	97.64
SA11	F422_gp153	273	9	90.72	Present (F422_gp078)	170	95.88
StAP1	SAP1_035	285	11	94.84	Present (SAP1_115)	170	95.88
Stau2	Stau2_17	255	6	84.19	Present (BH792_gp095)	170	95.88
ϕMR003	MRS_079	237	3	79.03	Present (MRS_154)	170	95.88
K	CPT_phageK_gp179	397	0	38.69	Present (CPT_phageK_gp115)	170	61.76
Twort	TwortORF029	323	0	39.57	Present (TwortDSMZ_157)	172	55.42

* Identity (%) was calculated using the protein maximum-maching program in GENETYX-Windows Ver.13

A search using GENETYX-Windows (ver. 13; Genetyx, Tokyo, Japan) also revealed that the 30-amino-acid N-terminal region of ORF10 is hydrophobic, indicating that it could be a membrane protein or a protein with a signal peptide. Although the PIEPEK repeat region was predicted by a BLASTp search to contain domains for TonB (periplasmic protein, link of inner membrane and outer membrane), IgG-blocking protein, and amelogenin (biomineralization), the function of ORF10 in the phage infection process remains unclear at present.

Another variable region was found between *orf88* and *orf89*. A specific 541-bp insert was found between *orf88* and *orf89* in KSAP11 only. An additional gene, *orf88.1*, was present in the inserted region. *Orf88.1* encoded a 170-amino-acid sequence identified as a putative tail protein including an Ig-like domain.

Phage KSAP7, which does not carry the *orf88.1* gene, was also able to grow in many *S. aureus* strains (Table S1), so ORF88.1 was considered to be additive but not essential for phage growth. A protein similar to ORF88.1 was also predicted in other phages belonging to the genus *Silviavirus* and in phages (K and Twort) belonging to different genera of the same subfamily (*Twortvirinae*) (Table 1)[8].

In addition, a single amino acid residue (glutamate) was also predicted to be added at the C-terminus in ORF88 in KSAP11 with insertion of the above sequence.

As there was a 50-bp direct repeat in the 3’region of both *orf88* and *orf88.1* (Fig. 2b), recombination between these repeat sequences might be related to the insertion/deletion of the sequence according to the Campbell model [18].

Here, we report two new members of the genus *Silviavirus* that appear to be suitable for phage therapy. A comparison of DNA sequences of members of the genus *Silviavirus*, including KSAP7 and KSAP11, suggested that phages in this genus may have at least two genomic regions where site-specific DNA rearrangements occur.

## Electronic supplementary material

Below is the link to the electronic supplementary material.

**Fig. S1** Phylogenetic relationship of KSAP7 and KSAP11 to other Silviaviruses. After the 5’ terminus of each phage DNA was aligned with that of phage Romulus, these DNA sequences were analyzed by the NJ method using MEGA7. (PPTX 298 kb)

Supplementary material 2 (XLSX 13 kb)
