# Supplementary material for: Characterization of two newly isolated Staphylococcus aureus bacteriophages from Japan belonging to the genus Silviavirus
Source: Arch Virol. 2020 Aug 3;165(10):2355–9. doi: 10.1007/s00705-020-04749-6 (PMC7497331; doi:10.1007/s00705-020-04749-6)
Supplement: Supplementary file 1 — Fig. S1 Phylogenetic relationship of KSAP7 and KSAP11 to other Silviaviruses. After the 5’ terminus of each phage DNA was aligned with that of phage Romulus, these DNA sequences were analyzed by the NJ method using MEGA7. (PPTX 298 kb) [file 705_2020_4749_MOESM1_ESM.pptx]

## Slide 1
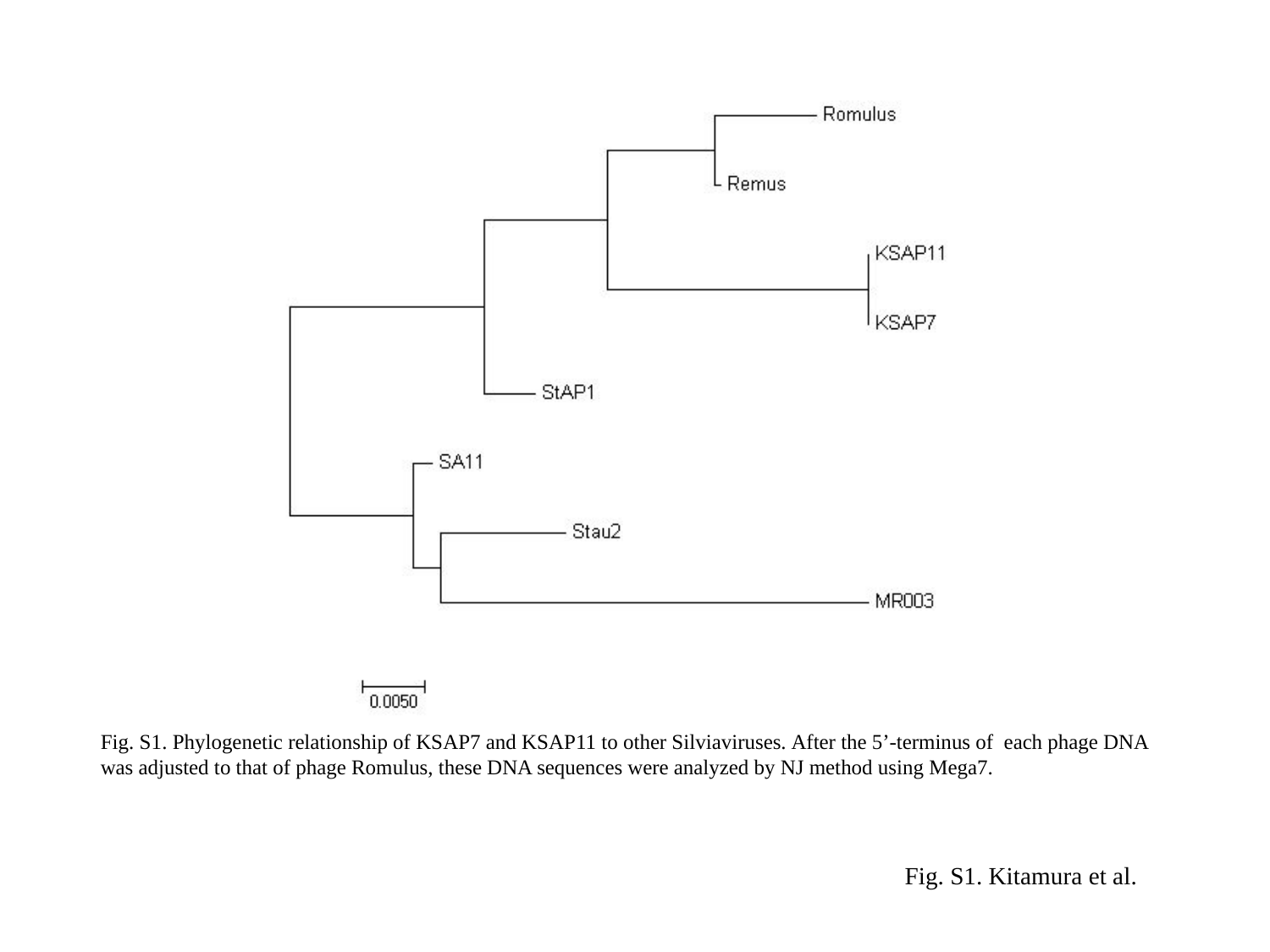

Fig. S1. Phylogenetic relationship of KSAP7 and KSAP11 to other Silviaviruses. After the 5’-terminus of each phage DNA was adjusted to that of phage Romulus, these DNA sequences were analyzed by NJ method using Mega7.
Fig. S1. Kitamura et al.
